# Human proprioceptive gaze stabilization during passive body rotations underneath a fixed head

**DOI:** 10.1038/s41598-024-68116-0

**Published:** 2024-07-29

**Authors:** Tobias Wibble, Tony Pansell

**Affiliations:** Division of Eye and Vision, Department of Clinical Neuroscience, Marianne Bernadotte Centrum, St. Erik’s Eye Hospital, Karolinska Institutet, Stockholm, Sweden

**Keywords:** Proprioception, Gaze-stabilization, Multisensory, Vestibular, Motion processing, Oculomotor system, Neurophysiology

## Abstract

The present study explored the presence of torsional gaze-stabilization to proprioceptive neck activation in humans. Thirteen healthy subjects (6 female, mean age 25) were exposed to passive body rotations while maintaining a head-fixed, gravitationally upright, position. Participants were seated in a mechanical sled, their heads placed in a chin rest embedded in a wooden beam while wearing an eye tracker attached to the beam using strong rubber bands to ensure head stability. The body was passively rotated underneath the head both in darkness and while viewing a projected visual scene. Static torsional gaze positions were compared between the baseline position prior to the stimulation, and immediately after the final body tilt had been reached. Results showed that passive neck flexion produced ocular torsion when combined with a visual background. The eyes exhibited rotations in the opposite direction of the neck’s extension, matching a hypothetical head tilt in the same direction as the sled. This corresponded with a predicted head rotation aimed at straightening the head in relation to the body. No such response was seen during trials in darkness. Altogether, these findings suggest that proprioception may produce a predictive gaze-stabilizing response in humans.

## Introduction

The process of gaze stabilization plays a crucial role in mitigating retinal slippage during motion^1^. This phenomenon has been extensively studied, partuclarly the vestibulo-ocular reflex (VOR) which is activated during head movements, and the optokinetic responses (OKR) which is by external visual stimuli^1,2^. These sensory systems, i.e., visual and vestibular input are crucial for motion perception, and also act as main contributors towards our sense of balance, and mismatched signals act as the primary contributor towards motion sickness or vertigo^3^. The third sense influencing balance is that of proprioception, which allows us to evaluate the position of our own body parts at any given time^4^. Both vision and vestibular input reliably lead to reflexive eye movements through gaze stabilizing reflexive arcs, the capacity for proprioception to induce similar responses in humans remains debated.

Animal trials in non-human primates have suggested that the neural sensitivity to proprioceptive input may be largest in the roll or pitch planes, involving a translational component to the neck tilt as compared to isolated rotations around the yaw axis^5^, and compared to movements in the pitch direction, roll plane motion produces gaze stabilizing reflexes that have a more nuanced gain, allowing a finely tuned evaluation of how the brain has processed motion information in its sensorimotor response^6–10^. Roll-plane gaze stabilization is principally comprised of two components, which may be seen in both the VOR and OKR: Ocular torsion and vertical vergence^11^. Visually induced ocular torsion exhibits notably lower magnitude compared to its vestibular counterpart, with a gain typically ranging between 1 and 4% relative to the visual rotation^7,12,13^. Nonetheless, this torsional response is susceptible to modulation by the density of visual cues within the visual scene, with heightened visual information correlating with an augmented torsional reaction^7,14^. The integration of visual and vestibular inputs is robust, as evidenced by shared cortical, subcortical, and cerebellar regions implicated in posture, self-motion control, and gaze stabilization^15–17^.

Several of the neural structures involved in visual and vestibular gaze stabilization in humans also share significant overlap with proprioceptive pathways^17,18^. In prior investigations, an examination involving trunk rotation while maintaining a stable head position on a headrest yielded no observable torsional response in a limited population of four subjects, suggesting that proprioceptive input does not contribute significantly to ocular torsion^19^, while other trials have produced small albeit recordable eye movements^20^. Conversely, contrasting findings emerged from subsequent research employing vibrational stimuli on neck muscles, which elicited a torsional response as evidenced by scleral search coil recordings conducted on individuals with unilateral vestibular deafferentation^21^. These disparate outcomes underscore the complexity of the interactions between proprioceptive signals and ocular motor control mechanisms, prompting further inquiry into the underlying physiological processes governing gaze stabilization. One may also note that the method of proprioceptive engagement through vibration of the neck muscles introduces a confounding factor in oscillations of the tissue mechanically activating the vestibular system; the vestibular activation has however been shown to be secondary to the proprioceptive signalling but nevertheless remains a confounding factor^22^. Considering the small gaze stabilizing amplitudes expected from a neck flexion a more ecological approach may be considered preferable. Such trials, involving neck tilts, have employed older eye tracking methodologies, relying on analysing images taken at 1-min intervals^19^, and significant advances in video eye tracking allows for much finer evaluations of gaze stabilization than was possible in previous studies. Such eye tracking has been used to evaluate vibration induced muscle activation^21^, or passive neck rotation in the yaw plane^23^, which may be less sensitive to multisensory integration than motor responses during roll rotations^5^. An investigation into the eye movement responses during neck flexion in the roll plane, implementing high frequency eye tracking, would consequently serve to elucidate the basic nature and presence of proprioceptive gaze stabilization in humans.

The present study aimed to evaluate the presence of a gaze stabilizing response to proprioceptive neck stimulations in the roll plane. This was done by performing passive full-body rotations of healthy participants while their heads remained fixed in a gravitationally upright position, causing a flexion of the neck. In order to evaluate the impact of visual information towards any recorded response, as recorded by a wearable video eye tracker, trials were carried out in both darkness and while viewing a visual scene.

## Material and methods

### Participants

Thirteen healthy volunteers (7 male, 6 female; mean age 25 (range 23–34)) were recruited for the study. None presented with any disorders or engaged in drug use known to impact the central nervous system. Each participant exhibited normal or corrected visual acuity (≥ 1.0 using LogMAR chart) and stereoscopic vision of at least 200 arcseconds (Lang II stereotest), alongside normal eye motility. The presence of latent strabismus was ruled out via the cover test. None of the participants reported a history of vertigo or any current neck injuries. Vestibular function was assessed as normal using the head impulse test, while balance was evaluated through the Romberg’s test.

Informed written consent was obtained from all participants prior to enrolment, following comprehensive explanation of the study’s nature and receipt of both written and oral procedural information. The research adhered to the principles of the Declaration of Helsinki and received approval from the Regional Ethics Committee of Stockholm (EPN 2018-1768-31-1).

### Experimental protocol

**Figure 1:**
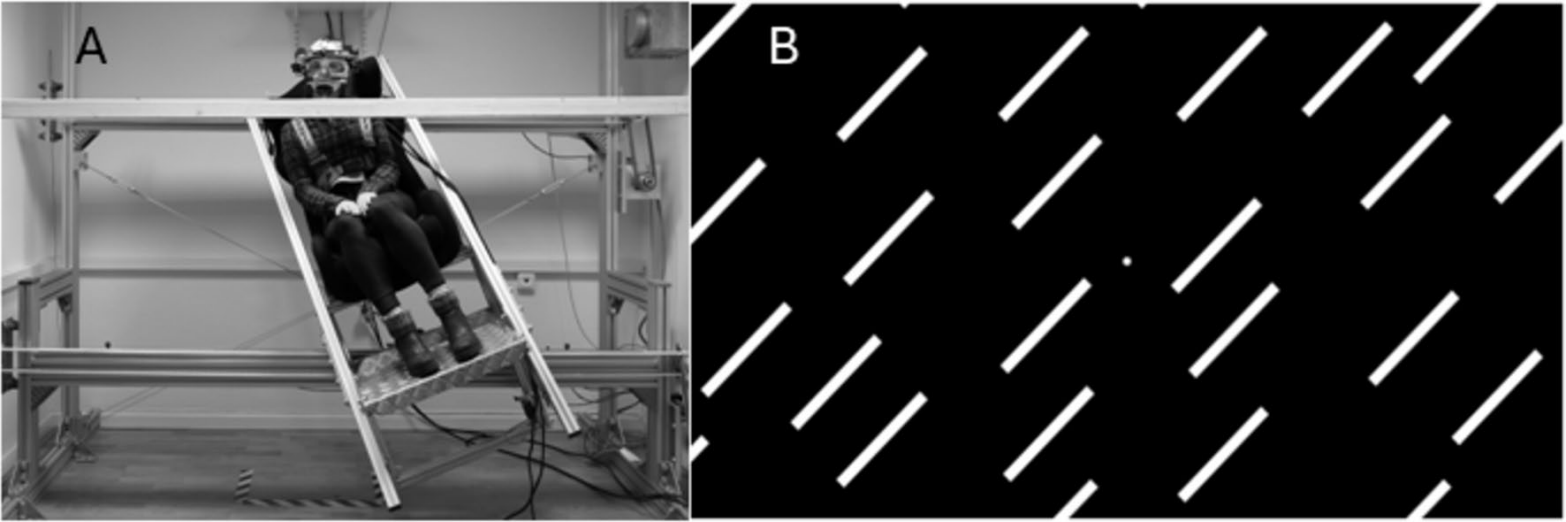
(**A**) Picture demonstrating passive trunk movement with the head fixed in place during proprioceptive stimulation in complete darkness, wearing an eye tracker (Chronos C-ETD). The head is fixed by a chinrest on a wooden beam secured to the wall. (**B**) Visual scene presented on the projector screen during the viewing condition. The scene consists of a central fixation point (0.32 cm in diameter) surrounded by 38 inclined white lines (0.42 cm long, 3.25 cm wide, visual angle 0.93 deg, tilted at 45 degrees).

Subjects were seated in a motorized sled in which they were exposed to whole-body rotations while maintaining a head-fixed position (Fig. 1A). In order to achieve head stability without risking injuries to the neck, subjects placed their chin in a chin-rest embedded in a wooden beam situated in front of the sled. Participants also wore a Chronos Eye-Tracking Device (C-ETD; Chronos Inc, Berlin), which in turn was attached to the beam through strong elastic bands. Proprioceptive stimulation was then performed through passive rotation of the body with subjects’ heads fixed in place, with the centre of rotation placed between subjects’ eyes, resulting in a neck rotation by lateral flexion. The wooden beam could also be manoeuvred vertically, allowing for adjustments for subjects’ height differences. Subjects’ trunks were then tilted by the mechanical sled to an amplitude of 14 degrees, which preliminary testing found to be tolerable while retaining a straight head without significant discomfort. This was carried out at 14 deg/s^2^ and 28 deg/s^2^ and repeated both in complete darkness and with subjects exposed to a projected visual scene (res 1024 × 768; contrast 2000:1; update frequency 60 Hz) at an eye-screen distance of two meters to present visually guided orientational cues (Fig. 1B). The sled rotation created an inverse pendulum effect where the head was stabilized in place and the trunk tilted beneath it, leading to proprioceptive stimuli being relayed through the neck.

In order to reduce the impact of learning effects due to repeated measurements, the test order was balanced between accelerations and modality (trials in darkness or viewing a projected screen) between subjects. Eye-and-head recordings were initiated 20 s before the active body-movement in order to allow for baseline values to be established. After the rotation of the body participants were kept at the tilted angle for an additional 20 s so that the static shift in gaze-position may be evaluated. Each trial was followed by a short break of ca 5 min to reduce the risk of neck strain.

### Eye and head movement recording

The motorized sled was synchronized with a head-mounted eye tracker, the C-ETD, which captured eye movements in three dimensions. This video-based eye tracker operated at a frame rate of 100 Hz and was integrated into a PC-based system with dedicated hardware and software. It allowed binocular tracking of horizontal and vertical eye movements with a spatial resolution of < 0.05 degrees, and enabled quantification of torsional eye movements via iris pattern recognition at a resolution of < 0.1 degrees. The latter methodology relied on template matching of individual frames with an initial reference frame acquired at the onset of recording. Utilizing cross-correlation, whereby a value of 1.0 indicates perfect matching, each frame was assigned a quality measure ranging between 0 and 1.0. Frames exhibiting a quality value below 0.5 were excluded from subsequent analysis to ensure data fidelity. Prior to measurements, eye movements were calibrated into degrees of rotation by recording eye positions at five predefined locations with known angular displacements. Additionally, the C-ETD included an integrated inertial head tracking system to monitor displacements and ensure control over head position in six dimensions (three rotational and three translational).

### Analyses

**Figure 2:**
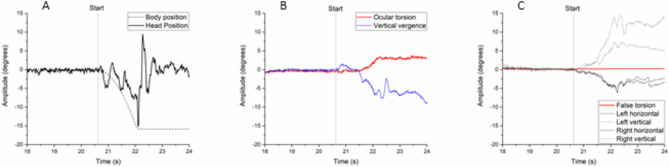
Illustrative traces for body and head positions in the roll plane (**A**), torsional, and vergence positions (**B**), and the horizontal and vertical positions for each eye during the stimulation, together with the false torsional response (**C**). The body position is inferred from the movement of the chair, which is recorded and synchronized together with the head-and-eye data. The false torsional response was calculated using the Fick-Helmholtz method, which was used to evaluate the artificial response for each eye and the false torsional position was subsequently retrieved as the average of these values. The stimulation start is indicated by a vertical line at 20,63 s as indicated by the start of the chair’s movement.

The torsional response was retrieved for both eyes and averaged to create a stable cyclotorsional value. The vertical vergence response was retrieved by deducting the right vertical eye position from the left vertical eye position. Both torsional and vergence values were plotted in Origin together with the roll-plane position of the chair and the head respectively (OriginPro 2017, OriginLab, Northampton, MA). The baseline position of torsion, vergence, and the head was smoothened using 25 points-of-window adjacent averaging to remove noise. Baseline positions were then collected by taking the mean positional values for each variable averaged over one second within the last three seconds prior to the stimulation; the timing was not kept constant due to irregularities such as blinks, and values were always retrieved when stable positions could be visualized. Due to the forces upon the head during the active movement of the sled, it was not possible to evaluate the gaze stabilizing responses with any desirable degree of certainty (Fig. 2), and the present study instead investigated the shift in static gaze-position to infer the gaze-stabilizing movement. Torsional, vergence, and head positions were, therefore, retrieved in the following seconds after the neck rotation according to the same principles as employed when ascertaining the baseline. Differences in the position of each variable were then calculated by subtracting the baseline position from the one observed after each trial. Still photos of the video eye recording were compared in order to preclude false recordings due to mask slippage (Appendix 1).

The statistical analyses were carried out using a Generalized Linear Model due to deviations from normality based, performed using IBM SPSS Statistics 25 for Windows, and the significance level (α) was set to 0.05. Two statistical analyses employing GLM was performed using linear scale distribution with a Wald Chi Square statistics: The first compared the raw eye position in degrees as the dependant variable, with factors included modality (passive neck rotation in darkness or while viewing a visual scene), acceleration (14 deg/s^2^ or 28 deg/s^2^), type of movement (head movement, ocular torsion, or vertical vergence), and time (before or after the passive neck rotation); a significant interaction effect involving time was considered obligatory for the passive neck stimulation to be considered as having a significant effect on gaze stabilization. Having established a significant effect, the second GLM implemented differences in amplitude as the dependant variable, keeping modality, acceleration, and movement type as factors. This was done to provide a clearer overview of the magnitude of the triggered eye movement response between subjects the starting eye position of the raw data may deviate due to slight differences in eye tracker placements; any effect on eye movement data was precluded through the calibration protocol outlined above.

### Informed consent

Informed consent was retrieved from all subjects for publication of identifying information/images in an online open-access publication.

## Results

All data was compiled in terms of that eye movement’s amplitudal shift between its baseline and the ocular position following the passive neck rotation. There were no missing data points, but due to poor signal quality of one subject’s torsional response the amplitude was retrieved eight seconds after termination of the stimulation for this trial. All other data retrievals were made within the first five seconds. Representative traces can be seen in Fig. 2.

In order to evaluate the risk of mask slippage as a confounding factor, correlational analysis was performed between the head position and the two eye movements. No significant correlation could be observed for neither torsion nor vergence, indicating that the two eye movements were observed independently of the head’s position as indicated by the mask. As any secondary torsion or vergence caused by mask slippage would have been directly associated with the position of the mask this meant that the risk for capturing artificial gaze-stabilizing was minimized. Furthermore, the still photos of the subject’s eye position before and after the stimulation can be found in Appendix 1, where comparing the positioning of facial features within the video frame supports the assumption of negligible mask slippage. In addition, degree of false torsion caused by horizontal and vertical eye movements were approximated according to the Fick-Helmholtz model^24^. These revealed negligible artificial torsion that were found to present both clockwise and counter-clockwise between trials, presenting no significant confounder for the interpretation of the data. For example, the torsional response for the subject in the representative trace was 3.58 degrees, while artificial torsion could account for 0.06 degrees (Fig. 2).

The statistical analysis evaluating raw eye position showed a significant interaction effect between time, modality, acceleration, and movement type (X^2^(4, N) = 14,633, *p* < 0.006), indicating that the stimulation provoked a gaze stabilizing response. The subsequent analysis of the difference in amplitude caused by the neck flexion revealed a significant interaction effect between modality and movement type (X^2^(2, N) = 7,915, *p* < 0.019). This meant that the change in position for each movement (head, torsion or vergence) was affected in different ways when the stimulation was carried out in darkness compared to when participants viewed a visual scene.

**Figure 3:**
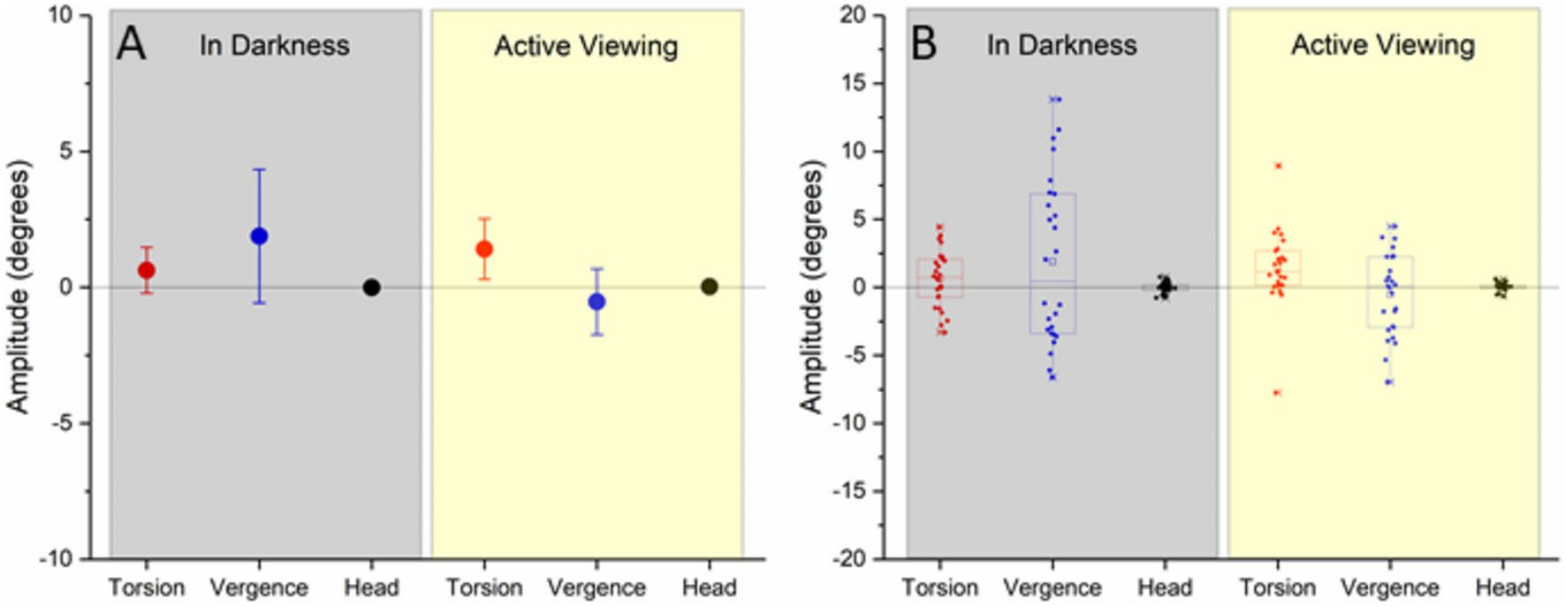
Changes in amplitude (degrees) for the torsional (red), vertical vergence (blue), and head position (black) before and after the passive neck rotation while subjects were head-fixed. Data is presented in interval plots (**A**) for readability, and as box charts (**B**) for a complete overview of the data. The interval plot illustrates the mean and 95% confidence interval, while the box chart shows the mean (square), median (horizontal line), 25–75% (rectangle), range within 1.5 interquartile range (whiskers) confidence interval (rectangle), and all data points with outliers given as stars. A reference line is given at the zero-mark on the y- axis. Trials carried out in darkness are highlighted by a translucent grey box, while trials carried out during the viewing condition are highlighted by a yellow box.

A one sample t-test showed that the torsional response observed during neck tilts with participants viewing a visual scene was significantly deviated from zero, indicative of a hypothetical head rotation contralateral to the neck flexion (t(25) = 2.605, *p* = 0.015; Fig. 3). The weak positive torsional value observed during body-rotations in darkness was however not significantly deviated from zero. Vertical vergence was also observed, presenting a positive value during trials in darkness, and a negative value during the viewing condition; it is expected that the vergence value be observed in the opposite direction to the torsional response, based on how the data is calculated, meaning that the pattern seen during trials in darkness is incongruous with an expected physiological response.

## Discussion

Gaze-stabilization serves to minimize retinal slippage during motion^1^. This is most often associated with self-generated head movements, activating the VOR, or when exposed to optokinetic stimuli, leading the eyes to pursue visual motion through the OKR^1,2^. These mechanisms rely on a few key subcortical structures, allowing basic sensorimotor integration in a way that has been largely conserved from the dawn of vertebrate life^25^. Proprioceptive contributions towards gaze stabilization remain more nuanced^26,27^. The lamprey, the oldest extant vertebrate, exhibits eye movements induced by self-generated locomotion, as do a range of more evolved vertebrates, including mammals^28^. The topic of human proprioceptive contributions towards gaze stabilization remains debated, with studies offering diverging conclusions on the matter^19,29,30^. From a neurophysiological standpoint, it is well-documented that the vestibular system, which is involved in both the VOR and OKR, has an important and complex interconnectivity with the musculature of the neck as has been shown in non-human primates and other vertebrates^31–33^. The neck receives vestibular afferents through the vestibulo-collic reflex (VCR), which leads to the neck reflexive adjusting its position in response to unexpected vestibular input or signals from its muscular stretch-receptors^32^. Inversely, the neck also sends afferents to the vestibular system and its interconnected network in the cerebellum^34^; these projections likely serve to allow for reflexive information on how the neck is positioned at any given time, allowing for the postural system to carry out its complex calculations^33^. There are neurons in the cerebellum which respond to both vestibular and proprioceptive stimuli, and which are silent when a movement is self-generated^34^, indicating that these neurons signal when proprioceptive input from the neck is incongruent with the expected head position as suggested by the peripheral vestibular organs. Inversely, self-generated head movements may be cancelled on a neuronal level should proprioceptive predictors in the neck suggest that the head movement agrees with the positioning of the neck; signals relaying the positioning of the neck has therefore been suggested to serve a gating function for further vestibular responses^31^.

The results found in the present study fits well within this context; proprioceptive data from stretch receptors in the neck aids in the prediction of the desired or expected head position, meaning that a gaze-stabilizing response may be expected in the predicted direction of a pending vestibular signal. It is noteworthy that proprioceptively induced ocular torsion was only observed in the presence of a viewed visual background. As the viewed scene was static, no confounding optokinetic motion may explain the eye movement response. The results therefore suggest that visual input, albeit static, serves as a directional cue that promotes proprioceptive input triggering a gaze-stabilizing response. Considering the neuronal basis for these eye movements it appears likely that this sensory integration occurs on a basic subcortical level. At this brainstem level, visual, vestibular, and proprioceptive input are well-integrated for the purpose of balance control^3^, and as this system shares several key integration points with the gaze-stabilization network one may expect that visuo-proprioceptive interactions would readily influence the reflexive eye movement response. One possible reason for trials in darkness failing to trigger ocular torsion may be that a lack of visual data indicates that no compensatory eye movement is needed; gaze-stabilization aims to minimize retinal slip, and the absence of visual input would indicate that any such adjustments are superfluous. In contrast however, the VOR causes reflexive eye movements even in darkness, albeit with increased gain during viewing conditions^14,35^. One may speculate that vision and proprioception may exhibit a similarly amplifying relationship, with vision acting as a signal enhancer, as the two senses are well integrated with that of the vestibular subcortical gaze-stabilizing network^5,32^. The present study summarily suggests that isolated proprioceptive signals may be insufficient to produce a significant eye movement response. This hypothesis could be tested in future studies by evaluating whether added visual information density would further enhance the torsional gain observed during visuo-proprioceptive trials.

Concerning the physiological purpose of the observed response, all participants experienced some level of discomfort while being placed at the tilted angle (Fig. 1A), wanting to straighten the head so that it may align with the body but failing to do so due to the mechanical constraints. The predicted head movement in such a scenario would arguably be one aiming to straighten the head, and the present investigation found that this type of torsional gaze stabilization was indeed observed during a static neck flexion. One may note that due to the limitations in head stabilization in the present study, results reflect the static gaze stabilizing response rather than the dynamic reflex. The change is amplitude caused by the neck flexion therefore serves as an indirect assessment of the ocular motor reflex, and while the setup failed to evaluate the eye movement gain in relation to the neck it allowed us to establish that proprioceptive gaze stabilization has a static component, likely priming the eyes for an expected head movement much like efference copies do in a range of vertebrate species^28^. The functionality of this response is nevertheless difficult to ascertain in the present study and would require further psychometric testing, evaluating if the response is an evolutionary remnant or retains a significant gaze stabilizing purpose.

Dealing with small amplitude eye movements invites some further limitations. For example, due to naturally occurring irregularities in the iris^36,37^ one can expect that the pupil size may cause confounding secondary displacements of the pupil, which the video system will misinterpret as eye movements. These are however quite small, and will always be in the same direction within each subject, meaning that directional values should balance out between individuals and trials. The present protocol also necessitates that we consider the risks of mask slippage as a confounder; if a subject were to rotate their head within the mask, the resulting eye movements would suggest a false movement of the head in the opposite direction to the head tilt, or ipsilateral to the flexion of the neck. This likely explains the irregular vergence responses observed in darkness (Fig. 3), as mask slippage would cause greater displacements in the vertical alignment compared to the torsional. Furthermore, while it was possible to ascertain the level of false torsion through the Fick-Helmholtz formula, it is more difficult to evaluate artificial vertical vergence. It would also be physiologically quite difficult for the eyes to diverge in such a way as were observed during the trials in darkness, as the direction should be inverse of the torsional response given how the vergence was calculated, i.e., an exotorsion would be congruent with a depression of the vergence response as the eye contralateral to a head tilt elevates in relation to the other eye. While the vergence response observed in the viewing condition appears more likely, its confidence interval crosses the zero marking (Fig. 3). We therefore suggest that the vergence response not be taken as indicative of gaze stabilization, though it may instead give an idea concerning the degree of mask slippage; the degree of this confounder should however be quite small as indicated by the still photos presented in Appendix 1. Torsion, in contrast, could be readily assessed, but the true response was likely somewhat diminished by the aberrant vergence activity in darkness. This should ever be negligible, and it consequently appears likely that the addition of visual information strengthened the predictive gaze stabilizing response. The study limitation of proper head fixations makes further inferences regarding visual and vestibular contributions towards the proprioceptive gaze stabilizing response precarious. Even minor head rotations, especially during the viewing condition, would lead to a gaze stabilizing response. One may speculate that the presence of vestibular and visual motion would contribute to the proprioceptive priming, as the brain receives additional information that movement is taking place. This would however have very little directional influence on the gaze stabilizing reflex as each minor head jolt was followed by a corresponding movement in the opposite direction (as seen in Fig. 2), leading to the head being placed straight at the end of the body rotation and a cancellation of vestibular and visual directional cues beyond the gravitational upright. Visual and vestibular input likely play an important role in the dynamic eye movement response during the neck flexion, and this relationship deserves further investigation in future studies. For example, carrying out trials using different levels of visual information density would allow for a more nuanced evaluation of the visual contribution, and with a better system for stabilizing the head one may investigate at what degree of flexion the proprioceptive response manifests, establishing the detection threshold.

It is well-documented that visual content enhances the gain of a gaze-stabilizing response^14,35^. It has also been shown that visual information will serve as important orientational landmark when assessing one’s own position in space^38^. The viewing condition therefore adds to the perception of tilt, as the position of the neck and body can be compared to both vestibular and visual perspectives of upright. While the impact of vision on neck proprioception in the yaw plane appears minor, an argument has been made for it having a stronger impact in the roll plane where horizontal translational components may also be involved^5,39^. The urge to stabilize the head may therefore be expected to become increasingly prominent in this condition, as both vision and proprioception encourage the desired head movement. Both visual and vestibular cues are relayed through joint vestibular mechanism on cortical, subcortical, and cerebellar levels^33^, and it appears quite plausible that these inputs may modify the proprioceptive gaze stabilizing response. It is also known that the human visual system is particularly sensitive to torsional disparities, as it is less adept at mitigating the breakdown of binocularity when faced with torsional disparities compared to those in the horizontal or vertical plane^40^. This sensitivity to roll plane disparities also offers some additional context concerning the physiological benefit of visual input enhancing a proprioceptive gaze-stabilizing signal caused by neck flexion, similar to its effect on the VOR^14^, as increasing the torsional gain decreases the amount of retinal slip. While it would be highly interesting and relevant to evaluate the dynamic component of the torsional eye movement, it was difficult to do so using the present protocol as it was not possible to maintain head stability during the active neck stimulation, and no further head stabilization was deemed suitable considering the possibility for neck injuries.

In conclusion, the present study showed that passive neck rotations underneath a fixed head may induce gaze stabilizing eye movements during viewing conditions. The direction of this response was predictive of an impending head movement aiming to align the neck with the body. This suggests that proprioceptive neck signals interact with static visual information to induce gaze stabilization in humans, with visual and proprioceptive input interacting to provide orientational cues for head-body alignment.

### Supplementary Information

Supplementary Figure 1.

Supplementary Legends.

## Data Availability

The datasets used and/or analysed during the current study available from the corresponding author on reasonable request.
