# Supplementary figures and images for: Human proprioceptive gaze stabilization during passive body rotations underneath a fixed head
Source: Sci Rep. 2024 Jul 29;14:17355. doi: 10.1038/s41598-024-68116-0 (PMC11286784; doi:10.1038/s41598-024-68116-0)

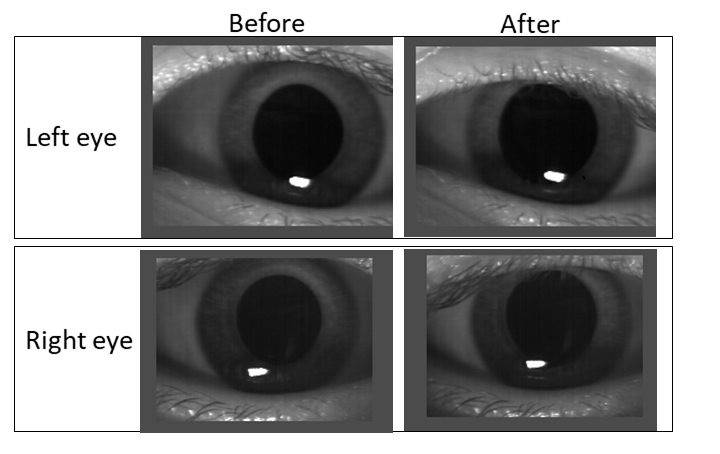

Supplement: Supplementary file 1 — Supplementary Figure 1. [file 41598_2024_68116_MOESM1_ESM.tif]
