# Supplementary material for: Human proprioceptive gaze stabilization during passive body rotations underneath a fixed head
Source: Sci Rep. 2024 Jul 29;14:17355. doi: 10.1038/s41598-024-68116-0 (PMC11286784; doi:10.1038/s41598-024-68116-0)
Supplement: Supplementary file 2 — Supplementary Legends. [file 41598_2024_68116_MOESM2_ESM.docx]

**Appendix 1.** Still photographs of the video used for the eye tracking analysis presented in figure 2. The stills are retrieved from 10s before and after the stimulation respectively, i.e., 10s into the recording and 30s into the recording. By comparing the positioning of the lower eyelid in relation to the camera one may conceptualize the extent of the mask slippage, as any significant mask slippage would have moved all facial features captured in the video. This figure consequently shows a negligible effect of mask slippage.
